# Association between cigar use, with and without cigarettes, and incident diagnosed COPD: a longitudinal cohort study

**DOI:** 10.1186/s12931-023-02649-2

**Published:** 2024-01-04

**Authors:** Steven Cook, James H. Buszkiewicz, David T. Levy, Rafael Meza, Nancy L. Fleischer

**Affiliations:** 1 Department of Epidemiology, University of Michigan, 1415 Washington Heights, Ann Arbor, MI 48109 USA; 2 Department of Oncology, Georgetown University, Washington, DC USA; 3 Department of Integrative Oncology, BC Cancer Research Institute, Vancouver, BC USA; 4 School of Population and Public Health, University of British Columbia, Vancouver, BC Canada

**Keywords:** Cigars, Cigarettes, COPD, Health effects

## Abstract

**Background:**

While regular cigar smoking is believed to carry similar health risks as regular cigarette smoking, the impact of cigar use, alone or in combination with cigarettes, on obstructive pulmonary disease (COPD) has not been well characterized. The purpose of this study was to examine the prospective association between exclusive and dual cigar and cigarette use and incident self-reported diagnosed COPD.

**Methods:**

This study used data from Waves 1–5 (2013–2019) of the Population Assessment of Tobacco and Health (PATH) Study, a nationally representative survey of U.S. adults. Longitudinal data from adults aged 40 to 79 at Wave 1, without a pre-existing COPD diagnosis who participated at follow-up interview were analyzed. A time-varying current tobacco exposure, lagged by one wave and categorized as: (a) never/non-current use; (b) exclusive cigar use; (c) exclusive cigarette use; and (d) dual cigar/cigarette use. Multivariable models adjusted for demographics (age, sex, race or ethnicity, education), clinical risk factors (asthma, obesity), and smoking-related confounders (second-hand smoke exposure, other combustible tobacco product use, e-cigarette use, time since quitting, cigarette pack-years). The incidence of self-reported diagnosed COPD was estimated using discrete-time survival models, using a general linear modeling (GLM) approach with a binomial distribution and a complementary log-log link function.

**Results:**

The analytic sample consisted of 9,556 adults with a mean (SD) age of 56 (10.4), who were predominately female (52.8%) and Non-Hispanic White (70.8%). A total of 906 respondents reported a diagnosis of COPD at follow-up. In the fully adjusted model, exclusive cigar use (adjusted hazard ratio (aHR) = 1.57, 95% CI: 0.77, 3.21) was not associated with increased COPD risk compared to non-use, while exclusive cigarette use (aHR = 1.48, 95% CI: 1.13, 1.93) and dual cigar/cigarette use (aHR = 1.88, 95% CI: 1.24, 2.85) were.

**Conclusions:**

Exclusive cigarette use and dual cigar/cigarette use were associated with diagnosed incident COPD. These results suggest that cigars, when used in combination with cigarettes, may be associated with poorer COPD health outcomes. Dual use may promote a higher likelihood of inhaling cigar smoke, and future research would benefit from examining whether inhalation of cigar smoke increases COPD risk.

**Supplementary Information:**

The online version contains supplementary material available at 10.1186/s12931-023-02649-2.

## Introduction

While United States (US) cigarette smoking prevalence among adults has steadily declined over the past two decades, cigar use has remained relatively stable [1]. US cigar sales increased between 2009 and 2020 [2], demonstrating the increasingly important role of cigars in the US tobacco marketplace. With 3.5% of US adults reporting current cigar use in 2020 [3], cigars represent a key public health concern, highlighting the need to better understand the health effects of cigar smoking [4].

Cigars contain many of the same hazardous constituents as cigarette smoke [5], and regular cigar smoking is believed to carry similar health risks as regular cigarette smoking [4–6]. A few studies have associated cigar use with lung dysfunction [7, 8], chronic obstructive pulmonary disease (COPD) [9, 10], and COPD-related mortality risk [4, 11]. However, the respiratory impact of cigar use is not well characterized as most adults who use cigars also use cigarettes, which many studies overlook. Moreover, there is a dearth of research examining the association between cigar use and COPD using longitudinal and population-based data.

Establishing an association between cigar use and COPD is complicated by the fact that the majority of adults who smoke cigars also smoke cigarettes [12], and cigarette smoking is a major risk factor for COPD [13–15]. The age-adjusted COPD prevalence of 14.1% among adults who currently smoke cigarettes and 7.1% among adults who formerly smoked cigarettes [16] demonstrates that a large proportion of COPD’s health and economic burden can be directly attributed to cigarette smoking [15]. Therefore, studies documenting the association between cigar smoking and COPD risk must adjust for cigarette smoking histories, including smoking status, duration, and intensity.

In this study, we examined the prospective association between exclusive and dual cigar and cigarette use and incident self-reported diagnosis of COPD among a nationally representative cohort of US adults. We (1) examined the risk of COPD prospectively; (2) developed a one-wave lagged exposure variable combining current cigar use and cigarette use to examine the relative contribution of exclusive cigar use and cigarette use and dual cigar/cigarette use to COPD risk, compared to non-use; and (3) adjusted for the duration, intensity, and history of cigarette smoking by adjusting for cigarette pack-years.

## Methods

### Study design and population

We used restricted-access data on adults from Waves 1–5 (2013–2019) of the Population Assessment of Tobacco and Health (PATH) Study, a nationally representative longitudinal study of the non-institutionalized civilian US population [17]. Details on the PATH Study are described elsewhere [18]. Information on accessing the restricted use data is described in the PATH Study Restricted Use Files User Guide at: 10.3886/Series606.21.

Consistent with the inclusion criteria from other national COPD cohort studies, we restricted the analytic sample to adult respondents aged 40 to 79 at Wave 1 (baseline) [19, 20] without a pre-existing COPD diagnosis (i.e., COPD, chronic bronchitis, or emphysema) who participated in at least one follow-up interview (Supplemental Fig. 1) [21]. We also excluded adults who exclusively smoked premium cigars for three reasons: compared to other types of cigar use, exclusive premium cigar use is (1) less likely to represent true established use [5], (2) less frequent (defined by the number of days used in the past 30 days) [22, 23], and (3) less likely to be used by adults who smoke cigarettes [22, 23]. These different usage patterns result in lower levels of toxicant exposure, potentially confounding the association between other types of cigar use and COPD [5].

### Measures

Self-reported diagnosed COPD incidence at each follow-up interview was based on the question: “*In the past 12 months, has a doctor, nurse, or other health professional told you that you had…(1) COPD, (2) chronic bronchitis, or (3) emphysema*?” Consistent with the clinical definition of COPD [24], respondents who reported being diagnosed with any of these conditions were considered to have COPD at follow-up.

Current cigar use was defined as established (ever used cigars fairly regularly) every day or someday use of traditional cigars, filtered cigars, or cigarillos in the past 30 days. Current cigarette use was defined as established (smoked 100 or more cigarettes in their lifetime) every day or someday use in the past 30 days. These two variables were then used to construct a four-category, time-varying cigar/cigarette exposure: (1) non-current use of either product (including never use and former use), (2) exclusive cigar use, (3) exclusive cigarette use, and (4) dual use of cigars and cigarettes. To ensure the cigar/cigarette exposure preceded the outcome, we lagged the time-varying exposure variable by one wave (*t*-1) and evaluated its association with the COPD outcome at wave (*t*).

Several baseline sociodemographic and health factors were included as covariates, including age (continuous), sex (male, female), race and ethnicity (Hispanic, non-Hispanic (NH) White, NH Black, another race and ethnicity), and education (high school or less, some college, bachelor’s degree/graduate degree). Obesity (body mass index (BMI) ≥ 30 vs. < 30), based on self-reported height and weight, and ever self-reported diagnosis of asthma were included as baseline COPD risk factors. Exposure to second-hand smoke (number of hours of ‘close’ exposure during the past seven days (range 0-100)) was included as a time-varying and lagged (*t-*1) COPD risk factor.

Several additional covariates were included to account for potential tobacco-related confounding. First, electronic nicotine delivery systems (ENDS) and ‘other’ combustible tobacco product use (pipes, hookah) were included as covariates. Both variables were based on current established use (ever use fairly regularly), and both variables were included as time-varying exposures, lagged by one wave (*t*-1). Second, using former established smoking (smoked at least 100 cigarettes in lifetime, but no current use) and years since quitting, a categorical time since quitting variable was constructed (non-former smoking, short-term quitting, long-term quitting). Non-former smoking includes never established and current smoking, while short-term quitting refers to former smokers who quit less than two years ago, and long-term smoking refers to former smokers who quit two or more years ago. Finally, cigarette pack-years were included to measure lifetime cigarette smoking at baseline. Cigarette pack-years were calculated by multiplying the reported years of cigarette smoking by the average number of packs per day while individuals smoked. Respondents who reported smoking more than 200 cigarettes daily (10 packs per day) were considered implausible and were set to missing (n = 99).

### Statistical analysis

Descriptive statistics were calculated for all variables at baseline. Tobacco-related variables were then examined according to respondent’s baseline cigar and cigarette use. Chi-square tests or Fisher’s exact tests were used to test for statistical differences between these groups. Lifetables were then generated to describe the distribution of self-reported diagnosed COPD.

Discrete-time survival models were used to examine the prospective association between exclusive and dual use of cigars and cigarettes and incident self-reported diagnosis of COPD at follow-up (Wave 2-Wave 5). These models provide the discrete-time equivalent to continuous time survival models [25], and we have used this methodological framework in previous health effects research [21, 26, 27]. Hazard ratios (HRs) and 95% confidence intervals (CIs) were fit using a general linear modeling (GLM) approach with a binomial distribution and a complementary log-log link function. All data were weighted using Wave 1 weights, including full-sample and 100 replicate weights, to ensure respondents represented the non-institutionalized US adult population at baseline. For all analyses, variances were computed using the balanced repeated replication methods with Fay’s adjustment set to 0.3, as recommended by the PATH study [28, 29]. All analyses were conducted using Stata 17.1 [30].

### Sensitivity tests

Several sensitivity analyses were conducted. First, to better understand how cigarette and cigar smoking histories impact COPD, we constructed a nine-category mutually exclusive cigarette/cigar exposure that disaggregated non-use into never and former use: (1) never cigarette or cigar use, (2) never cigarette use, former cigar use, (3) never cigarette use, current cigar use, (4) former cigarette use, never cigar use, (5) current cigarette use, never cigar use, (6) former cigarette use, former cigar use, (7) former cigarette use, current cigar use, (8) current cigarette use, former cigar use, (9) current cigarette use, current cigar use. Second, to assess sensitivity to the choice of PATH survey weights, ‘wave 2 weights,’ which restricted the analysis to respondents who participated in the first follow-up interview, and the ‘all waves weights,’ which restricted the analysis to the longitudinal cohort of respondents who participated in all waves of the PATH study were both considered. To test the impact of our decision to remove adults who used exclusive premium cigars from the analytic sample, discrete-time models were estimated, including these respondents. Finally, because research suggests that COPD may be prevalent among younger adults [31], we expanded our analytic sample to include adult respondents aged 25–39.

## Results

At baseline, sample respondents ($n$=9,559) had a mean age of 56 years (SD = 10.4) and were predominately female (52.8%), NH White (70.8%), and with some postsecondary education (61.4%) (Table 1). Approximately one-third of respondents had a BMI of 30 kg/m^2^ or more (34.3%), and 8.9% reported a lifetime diagnosis of asthma at baseline. Most respondents did not have current cigar or cigarette use at baseline (85%). Of those, 2.2% had short-term former cigarette use, and 20.6% had long-term former cigarette use. Among respondents with current or former established cigarette smoking, the average cigarette pack-years at baseline was 25.9 (SD = 26.3). Regarding exposure to second-hand smoke, 42.4% reported past 7-day exposure to second-hand smoke, with an average 10.2 h (SD = 22.1) of exposure. At baseline, 13.6% of respondents (n = 3,173) reported exclusive cigarette use, while exclusive cigar use and dual cigar/cigarette use was reported by 0.6% (n = 134) and 0.8% (n = 184) of respondents, respectively.

**Table 1:** Sample characteristics for analytic sample, PATH Study (Wave 1, 2013–2014)

	N	%^a^	95% CI
Age (mean, sd)	9,559	56.0 (10.4)	
Sex			
Female	4,743	52.8	52.0, 53.7
Male	4,816	47.2	46.3, 48.0
Race/Ethnicity			
NH White	6,331	70.8	69.8, 71.7
Hispanic	1,188	11.5	10.8, 12.4
NH Black	1,505	11.5	10.9, 12.1
NH Other	535	6.2	5.7, 6.8
Education			
High School or Less	3,963	38.6	37.9, 39.4
Some College	3,069	29.5	28.8, 30.2
Bachelor or Higher	2,527	31.9	31.2, 32.6
*Baseline Risk Factors*			
Asthma Diagnosis			
No	8,715	91.1	90.3, 91.7
Yes	844	8.9	8.3, 9.7
Obesity (BMI > = 30)			
No	6,278	65.7	64.4, 67.0
Yes	3,281	34.3	33.0, 35.6
*Tobacco Product Exposure*			
ENDS^b^ use			
No	9,203	98.5	98.3, 98.7
Yes	356	1.5	1.3, 1.7
Other’ combustible product use^c^			
No	9,460	99.6	99.4, 99.6
Yes	99	0.4	0.36, 0.56
Cigarette Smoking Status			
Non-former smoker	7,720	77.2	75.8, 78.4
Short-term former smoker	334	2.2	1.9, 2.6
Long-term former smoker	1,523	20.6	19.4, 21.9
*Cigarette and Cigar Use*			
Non-Current Use	6,068	85	84.3, 85.6
Exclusive Cigarette Use	3,173	13.6	13.0, 14.3
Exclusive Cigar Use	134	0.64	0.52, 0.74
Dual Use	184	0.76	0.65, 0.89
Pack years among currently/formely smoking (mean, sd)	5,214	25.9 (26.3)	
Past 7 day second hand smoke exposure			
No	3,971	57.6	56.1, 59.2
Yes	5,588	42.4	40.8, 43.9
Past 7 day second hand smoke exposure (mean, sd)	5,588	10.2 (22.1)	

^a^ percentages were calculated using Wave 1 weights

^b^ ENDS = electronic nicotine delivery systems

^c^ ‘Other’ combustible tobacco product use consisted of pipe and hookah use

Compared to adults who exclusively smoked cigars, adults who smoked cigarettes exclusively or in combination with cigars had higher levels of second-hand smoke exposure (Table 2). Among adults who smoke cigars, traditional cigars were more likely to be used (54.7%) and were used on more days during the past month (x = 9.6) by those who exclusively smoke cigars than by those who smoke cigars and cigarettes (36.4%; x = 5.2). In contrast, filtered cigars were more likely to be used by adults who smoke cigars and cigarettes (55.9%) and were used on more days during the past month (x = 11.0) than those who smoke cigars exclusively (27.4%; x = 5.9). Cigarillos (48.5% vs. 44.8%) and poly cigar types (27.3% vs. 29.7%) were not statistically different between the two groups of cigar use.

**Table 2:** Tobacco-related variables by established cigar/cigarette use at baseline, PATH Study (Wave 1, 2013–2014)

	Exclusive Cigarettes (n = 3173)		Exclusive Cigars (n = 134)		Dual Cigars/Cigarettes (n = 184)		Sign
	%^a^	95% CI	%^a^	95% CI	%^a^	95% CI	*P* ^b^
*Tobacco Variables*							
ENDS^c^ use	6.2	(92.7, 94.7)	6.4	(3.1, 12.8)	8.1	(4.7, 13.6)	NS
’Other’ combustible tobacco product used^d^	0.7	(0.44, 1.1)	9.1	(5.0, 16.0)	9.6	(6.1, 14.9)	***
Past 7 day second hand smoke exposure (mean, sd)	15.9 (37.0)		10.7 (29.5)		20.6 (43.5)		***
Cigarette pack years among currently/formerly smoking (mean, sd)	25.5 (32.8)		9.5 (21.0)		30.5 (37.6)		***
*Cigar Type*							
Traditional Cigars	---	---	54.7	(46.2, 62.9)	36.4	(29.7, 43.8)	**
Filtered Cigars	---	---	27.4	(20.0, 36.4)	55.9	(47.9, 63.6)	***
Cigarillos	---	---	48.5	(39.1, 58.0)	44.8	(38.1, 51.8)	NS
Poly cigar types used	---	---	27.3	(20.0, 36.1)	29.7	(22.7, 37.8)	NS

^a^ percentages were calculated using Wave 1 weights

^b^ Indicates statistical significance; NS = not statisticallly significant, *p < 0.05, **p < 0.01, ***p < 0.001

^c^ ENDS = electronic nicotine delivery systems

^d^ ‘Other’ combustible tobacco product use consisted of pipe and hookah use

A total of 906 of the 9,559 respondents in the analytic sample reported a COPD diagnosis at follow-up (Table 3). The weighted conditional probability of a COPD diagnosis ranged between 1.4% and 2.4%, with an average incidence of 2% during the five years of follow-up.

**Table 3:** Life tables describing the incidence of self-reported COPD, PATH Study (Waves 1–5, 2013–2019)

Interval	Total	COPD Diagnosis	Censored	Hazard Estimate^a^
Period 1 (W1-W2)	9,559	308	578	0.024
Period 2 (W2-W3)	8,673	243	687	0.0197
Period 3 (W3-W4)	7,743	154	732	0.014
Period 4 (W4-W5)	6,857	201	6,656	0.0197

^a^ hazard estimates were calculated with the replicate weights

The results from discrete-time models examining the association between the cigar/cigarette exposure and incident COPD outcome are presented in Table 4. When compared to non-current use in the unadjusted model (Model 1), exclusive cigarette use (hazard ratio (HR) = 2.91, 95% CI: 2.45, 3.33), and dual cigar/cigarette use (HR = 3.73, 95% CI: 2.61, 5.30) were both associated with an increased risk of incident COPD risk, while exclusive cigar use was not (HR = 1.29, 95% CI: 0.65, 5.30). After adjusting for sociodemographic risk factors, baseline risk factors, and tobacco-related variables, the point estimates were reduced for exclusive cigarette and cigarette/cigar use, but the substantive interpretation remained the same. In the multivariable model (Model 4), both exclusive cigarette use (adjusted hazard ratio (aHR) = 1.48, 95% CI: 1.13, 1.93), and dual cigar/cigarette use (aHR = 1.88, 95% CI: 1.24, 2.85) were associated with increased COPD risk compared to non-current use, while exclusive cigar use was not (aHR 1.57, 95% CI: 0.77, 3.21).

**Table 4:** Discrete-time survival analysis predicting self-reported COPD incidence, PATH Study (Waves 1–5, 2013–2019)

	Model 1^a^		Model 2^b^		Model 3^c^		Model 4^d^	
	Hazard	95% CI	Hazard	95% CI	Hazard	95% CI	Hazard	95% CI
*Time-Varying Cigarette and Cigar Use*								
Non-Current Use	REF	REF	REF	REF	REF	REF	REF	REF
Exclusive Cigarette Use	2.91***	2.45, 3.33	2.78***	2.27, 3.41	2.85***	2.32, 3.49	1.48**	1.13, 1.93
Exclusive Cigar Use	1.29	0.65, 2.56	1.54	0.76, 3.13	1.5	0.73, 3.11	1.57	0.77, 3.21
Dual Use	3.73***	2.61, 5.30	3.60***	2.53, 5.14	3.69***	2.52, 5.39	1.88**	1.24, 2.85
Age^e^			1.04***	1.02, 1.04	1.04***	1.03, 1.05	1.03***	1.02, 1.04
Sex (Female = 1)			1.59***	1.35, 1.93	1.60***	1.32, 1.94	1.72***	1.43, 2.07
Race/Ethnicity								
NH White			REF	REF	REF	REF	REF	REF
Hispanic			1	0.78, 1.28	1.03	0.81, 1.32	1.18	0.92, 1.50
NH Black			1.08	0.86, 1.35	1.11	0.88, 1.40	1.21	0.95, 1.54
NH Other			1.03	0.65, 1.62	1.05	0.65, 1.65	1.08	0.68, 1.71
Education								
High School or Less			1.94***	1.51, 2.51	1.93***	1.50, 2.50	1.74***	1.33, 2.27
Some College			1.46*	1.09, 1.95	1.45*	1.09, 1.94	1.34*	1.0, 1.80
Bachelor Degree or Higher			REF	REF	REF	REF	REF	REF
*Baseline Risk Factors*								
Asthma			2.93***	2.28, 3.76	2.91***	2.27, 3.75	3.0***	2.31, 3.90
Obesity (BMI > 30)			1.42***	1.19, 1.71	1.41***	1.18, 1.70	1.40***	1.17, 1.68
Time-varying second-hand smoke exposure^f^			1.10***	1.06, 1.15	1.11***	1.06, 1.15	1.08**	1.03, 1.13
Time-varying ‘other combustible’ use^g^					1	0.37, 2.63	0.95	0.35, 2.62
Time-varying ENDS^h^					1.26	0.88, 1.81	1.14	0.80, 1.65
Cigarette Smoking Status								
Non-former smoker					REF	REF	REF	REF
Short-term former smoker					1.3	0.81, 2.08	0.68	0.40, 1.16
Long-term former smoker					1.21	0.92, 1.60	0.64*	0.45, 0.91
Log cigarette pack-years^f^							1.93***	1.63, 2.28

Notes: Person N = 9,559; risk N = 32,832; *p < 0.05, **p < 0.01, ***p < 0.001

^a^ cigar/cigarette exposure ^b^ sociodemographic variables added; ^c^ smoking status added; ^d^ cigarette pack-years added

^e^ age mean centered for models

^f^ variable rescaled to reflect intervals of 10

^g^ ‘Other’ combustible tobacco product use consisted of pipe and hookah use

^h^ ENDS = electronic nicotine delivery systems

### Sensitivity analyses

Compared to never established use of cigars or cigarettes, current cigarette use/never cigar use (aHR 1.61, 95% CI 1.17, 2.23) and dual cigar/cigarette use (aHR 2.06, 95% CI 1.31, 3.24) were associated with increased risk of incident COPD in the multivariable model (Table S1, Model 3). Discrete-time models were also estimated using PATH Wave 2 weights (Table S2) and PATH “all-waves weights” (Table S3); with exclusive premium cigar use included in the analytic sample (Table S4) and with the analytic sample extended to include adult respondents aged 25 to 79 at baseline (Table S5). Across all sensitivity analyses, the substantive results remained consistent: exclusive cigar use was not associated with incident COPD risk, while exclusive cigarette and dual cigar/cigarette use were.

## Discussion

Using nationally representative data from the PATH cohort study, we examined the prospective association between cigars, cigarettes, and incident COPD among US adults aged 40 to 79. We found that time-varying exclusive cigarette use and baseline cigarette pack-years were both associated with increased incident COPD, which was expected since current cigarette smoking [32] and cumulative smoking [14, 33, 34] are major COPD risk factors. Consistent with other cigar research [1, 12], most adults with established cigar use had established histories of cigarette smoking, time-varying dual use of cigars and cigarettes was associated with an increased risk of incident COPD compared to non-current use in models accounting for current cigarette use and cigarette pack-years. However, we did not find an association between time-varying exclusive cigar use and incident COPD risk compared to non-current use.

Adults with dual cigar/cigarette use had different tobacco-related use profiles than adults who used cigars or cigarettes exclusively. Compared to the exclusive use of either product, people who used cigars and cigarettes had higher levels of second-hand smoke exposure and higher cigarette pack-years. While these risk factors are important for determining whether tobacco use will lead to COPD [33, 35], adjusting for these tobacco-related differences did not attenuate the association between dual cigar/cigarette use and incident COPD in multivariable models. This suggests that factors other than cigarette smoking histories may be salient. For instance, while the small number of adults with current established cigar use in our study limited our ability to disaggregate cigar use by cigar type in models, filtered cigar use was more prevalent and smoked more frequently by adults with dual cigar/cigarette use. By contrast, traditional cigar use was more prevalent and smoked more frequently by adults with exclusive cigar use. Filtered cigars have been marketed as an inexpensive cigarette substitute [36], and associated with higher levels of cigar smoking frequency and intensity [22]. It is possible that the dual cigar/cigarette association is driven by higher levels of exposure based on differences in cigar type use, and examining the health effects separately by cigar type should be considered in future research. Conversely, inhalation of cigar smoke is known to be a key determinant of cigar-related health outcomes [4], and adults who smoke cigarettes are more likely than cigarette naïve cigar smokers to inhale cigar smoke [4, 37]. It is also possible that the dual cigar/cigarette association may be driven by the inhalation of cigar smoke by adults who smoke cigarettes; cigar inhalation behavior should also be considered in future research on the health effects of cigar use.

We did not observe an association between exclusive cigar use and incident COPD over the 5-year follow-up period. This finding is consistent with the results from another recently published study using PATH data [38]. In this study, Paulin et al. examined the association between 12 categories of current tobacco product use and COPD, and found that exclusive past 30-day cigar use (*n* = 391) was not associated with incident COPD risk compared to adults who never used tobacco [38]. However, in our study, while exclusive cigar use was not statistically associated with COPD risk, we observed an elevated hazard ratio compared to non-current use (aHR = 1.57) that was similar in magnitude to the hazard ratio for exclusive cigarette use (aHR = 1.48), albeit not statistically significant. The wide confidence intervals associated with the exclusive cigar use hazard ratio (95% CI 0.77, 3.21) suggests a lack of power, which is important to note given our exclusive cigar use sample was small (*n* = 130 at baseline). The main reason our sample of exclusive cigar users was smaller than the sample in the Paulin et al. study was because we excluded respondents who exclusively smoked premium cigars from our analytic sample because they are used less frequently than other cigar types and are less likely to represent true established use [5]. In a sensitivity analysis where exclusive premium cigar users were included in the analytic sample (Table S5), the number of respondents in the exclusive cigar use group increased, but the hazard ratios were lower (unadjusted HR = 0.97; fully adjusted HR = 1.29). Since exclusive premium cigar use is likely to have a different COPD risk profile compared to other types of cigar use, it is important to distinguish premium use from non-premium use.

Cigars and cigarettes are known to emit similarly harmful chemical constituents [5], and there is strong biological plausibility that exposure to regular cigar use, particularly if the smoke is inhaled, will be associated with increased risk of deleterious health outcomes [5], including increased COPD risk [9, 10]. In this study, the lower levels of COPD risk observed among people with exclusive cigar use compared to dual use is likely due to the lower frequency of use among exclusive cigar users, which results in less regular exposure to the harmful constituents of cigar smoke compared to dual use. It remains likely that frequent cigar use, especially when the cigar smoke is inhaled, will increase COPD risk independent of cigarette smoke. With the expansion of the cigar market in the US, future studies with greater statistical power are needed to evaluate the COPD risk associated with exclusive cigar use.

There are several important limitations to note. First, the results from this study are based on self-reported incident diagnosed COPD rather than on results from a spirometry test. This is a limitation of the PATH data. Still, the COPD prevalence estimates in PATH are generally consistent with those from the National Health and Nutritional Examination Survey [38]. Prior research has also demonstrated concurrent validity with other self-reported PATH health outcomes [39]. Second, PATH does not collect information on the level of cigar use inhalation, which has been strongly associated with negative health outcomes [5]. Future iterations of PATH would benefit from including inhalation questions for adults who smoke cigars. Third, the current research included cigarette pack-years as a measure of lifetime cigarette use but did not include an analogous measure of lifetime cigar use. Developing a standardized measure of lifetime cigar use is an important task for future research as it would allow for an estimate of lifetime frequency and intensity of cigar use on respiratory health. Fourth, the few respondents using cigars at each wave may have limited power to detect an association between exclusive cigar use and COPD. Moreover, the relatively small sample of adults with established cigar use meant it was not possible to disaggregate traditional cigars from cigarillos and filtered cigars. Given the important differences across cigar types [22, 23], future research would benefit from looking at the respiratory health effects of filtered cigar use separately from cigarillos and traditional cigars. Larger, more diverse cohorts that oversample adults who use cigars may be able to build on our analyses and further examine the complexities of cigar use behavior.

## Conclusions

In summary, our longitudinal analysis of the prospective association between cigar and cigarette use and incident diagnosed COPD provides evidence that exclusive cigarette use and dual cigar/cigarette use were associated with increased COPD risk. These findings remained statistically significant after controlling for confounders, including cigarette pack-years, providing evidence that cigar use, especially combined with cigarettes, is an independent risk factor for COPD. As cigars continue to play an increasingly important role in the US tobacco marketplace, our results demonstrate the need to implement policies designed to reduce the prevalence of cigar use.

### Electronic supplementary material

Below is the link to the electronic supplementary material.

Supplementary Material 1

## Data Availability

The current study used restricted use data from the PATH Study because the analysis included cigarette pack-years and continuous age. Information on accessing the restricted use data is described in the PATH Study Restricted Use Files User Guide at: 10.3886/Series606.21.
