# Correction to: Association between cigar use, with and without cigarettes, and incident diagnosed COPD: a longitudinal cohort study

**DOI:** 10.1186/s12931-024-02728-y

**Published:** 2024-02-23

**Authors:** Steven Cook, James H. Buszkiewicz, David T. Levy, Rafael Meza, Nancy L. Fleischer

**Affiliations:** 1 Department of Epidemiology, University of Michigan, 1415 Washington Heights, Ann Arbor, MI 48109 USA; 2 Department of Oncology, Georgetown University, Washington, DC USA; 3 Department of Integrative Oncology, BC Cancer Research Institute, Vancouver, BC USA; 4 School of Population and Public Health, University of British Columbia, Vancouver, BC Canada

**Correction to: Cook et al. Respiratory Research (2024) 25:13**

10.1186/s12931-023-02649-2

Following publication of the original article [1], the author’s name James H. Buszkiewicz was incorrectly written as James Buskiewicz.

The author group has been updated above and the original article [1] has been corrected.

## References

[1] Cook, S., Buskiewicz, J.H., Levy, D.T. et al. Association between cigar use, with and without cigarettes, and incident diagnosed COPD: a longitudinal cohort study. Respir Res. 2024;25:13. 10.1186/s12931-023-02649-2. 10.1186/s12931-023-02649-2 PMC10765880 38178199

