# Supplementary material for: Association between cigar use, with and without cigarettes, and incident diagnosed COPD: a longitudinal cohort study
Source: Respir Res. 2024 Jan 4;25:13. doi: 10.1186/s12931-023-02649-2 (PMC10765880; doi:10.1186/s12931-023-02649-2)
Supplement: Supplementary file 1 — Supplementary Material 1 [file 12931_2023_2649_MOESM1_ESM.docx]

Supplemental Material

Figure S1. Flowchart of Sample Selection for Analytic Sample

13,825 adult respondents aged 40-79 participated in the W1 survey

1951 baseline respondents did not respond to an of the follow-up waves (W2-W5)

11,874 adult respondents aged 40-79 at baseline completed at least one wave of follow up

1,401 respondents had a COPD outcome (COPD, chronic bronchitis, emphysema) at baseline

10,473 adult respondents aged 40-79 completed at least one follow-up wave and did not have COPD diagnosis at baseline

17 respondents participated in at least one follow-up interview but did not respond to COPD questions

10,456 adult respondents aged 40-79 at baseline participated at follow-up and reported information about COPD

111 premium exclusive cigar smokers at baseline were excluded

10,345 adult respondents aged 40-79 participated at follow-up, had follow-up information and were not exclusive premium cigar smokers

786 respondents had missing information on independent variable(s) for the main analysis

9,559 adult respondents aged 40-79 at baseline who particpated at follow-up and had complete information

Table S1. Discrete-time survival analysis predicting self-reported chronic obstructive pulmonary disease (COPD) incidence with **nine-category cigarette/cigar exposure** for respondents aged 40-79, Population Assessment of Tobacco and Health Study (Waves 1-5, 2013-2019).

Table S2. Discrete-time survival analysis predicting the incidence of self-reported chronic obstructive pulmonary disease (COPD) **using Wave 2 weights** for respondents aged 40-79, Population Assessment of Tobacco and Health Study

Table S3. Discrete-time survival analysis predicting the incidence of self-reported chronic obstructive pulmonary disease (COPD) **using "all-waves weights"** for respondents aged 40-79, Population Assessment of Tobacco and Health Study

Table S4. Discrete-time survival analysis predicting the incidence of self-reported chronic obstructive pulmonary disease (COPD) **with exclusive premium cigar use included** for respondents aged 40-79, Population Assessment of Tobacco and Health Study (Waves 1-5, 2013-2019)

Table S5. Discrete-time survival analysis predicting the incidence of self-reported chronic obstructive pulmonary disease (COPD) **for respondents aged 25-79**, Population Assessment of Tobacco and Health Study (Waves 1-5, 2013-2019)
